# Women in Science: Bridging Gaps in Basic Sciences

**DOI:** 10.1016/j.molmed.2025.07.005

**Published:** 2025-08-11

**Authors:** Bhavita Kumari, Narjis Fatima Hussain, Stephen H. Kennedy, Aris T. Papageorghiou, Jai K. Das, Zulfiqar A Bhutta

**Affiliations:** 1 Institute for Global Health and Development, The Aga Khan University, Karachi, Pakistan.; 2 Centre for Global Child Health, The Hospital for Sick Children, Toronto, Ontario, Canada.; 3 Oxford Maternal and Perinatal Health Institute, University of Oxford, Oxford, United Kingdom; 4 Nuffield Department of Women’s and Reproductive Health, University of Oxford, United Kingdom; 5 Department of Pediatrics and Child Health, The Aga Khan University, Karachi, Pakistan.

**Keywords:** Capacity-Building, Mentorship, Gender Disparities, Gender Equity in Research, Research Leadership

## Abstract

The Supporting Women in Science (SWIS) program aims to strengthen female representation in research in developing countries. This article highlights the program participation, mentorship challenges, and systemic barriers. Findings inform inclusive, discipline-specific strategies designed to support women’s research capacity, academic leadership, and advancement in the global scientific community.

## Advancing Gender Equity in Academia

Gender disparities in academia persist worldwide, with particularly stark gaps in low- and middle-income countries (LMICs) [1]. According to United Nations Educational, Scientific and Cultural Organization (UNESCO), women comprise less than 30% of researchers globally, with even lower proportions in South and West Asia (19%) and East Asia and the Pacific (24%) [2]. These inequalities hinder progress toward Sustainable Development Goals (SDGs) 4 and 5, that is, Quality Education and Gender Equality. Despite increasing female participation in research, women continue to publish less and secure fewer international collaborations than men [3].

In high-impact journals, female authorship remains disproportionately low, with a notable negative correlation between journal impact factor and female representation [4]. Challenges related to publication further impede women’s career advancement, as they are less likely to be first authors in high-impact journals [5]. Research funding disparities persist as women receive fewer grants and lower amounts due to bias in the review process [6]. A study of Canadian Institutes of Health Research grants revealed significantly lower success rates for female applicants, highlighting possible bias in the grant review process [7]. In Canada, only 63% of female faculty members held tenured positions compared to 75% of men, and women expressed greater dissatisfaction with hiring and promotion fairness [8]. Institutional biases related to hiring practices, promotion, and the tenure process further widen gender gaps, resulting in women receiving lower salaries and fewer tenure-track opportunities [9]. Basic sciences have some of the highest ratios of gender inequities favoring men, often more than clinical fields or public health [3]. This is attributed to long-standing perceptions as being male-dominated, lower visibility of female role models, and fewer targeted interventions addressing equity within these disciplines in LMICs [9].

Addressing these inequities requires targeted interventions such as gender-inclusive policies, equitable funding, mentorship initiatives, and institutional commitment to diversity in science. Systemic reforms can enhance women’s representation and contributions in basic sciences, ensuring equal opportunities for career advancement [10]. The Supporting Women in Science (SWIS) program, developed by institutions in Pakistan, Canada, and the UK, aims to bridge this gap by providing research capacity development for early-to mid-career female scientists in South-Central Asia and East Africa. The initiative builds on the strength of existing global research networks to empower women, enhance their scientific contributions, and promote gender equality in academia. This article provides a critical review of the SWIS initiative and offers an empirical reflection on female representation in research programs in LMICs and highlights the key insights from the SWIS program. This article underscores the discipline-specific participation trends, identifies challenges in mentorship and capacity-building and highlights actionable strategies to strengthen inclusion of early career women researchers.

## Representation of basic sciences in the SWIS program

The program is structured into three phases. Phase I involves the nomination of early-to-mid-career female researchers for a tiered capacity-strengthening initiative, which includes high-quality, self-paced online coursework. Participants who successfully complete Phase I requirements are invited to Phase II, which is a summative assessment and serves as a shortlisting criterion for the next phase. In Phase III, fellows receive research grants under remote mentorship from prominent faculty at Aga Khan University (AKU), University of Oxford and other leading universities.

**Figure 1:**
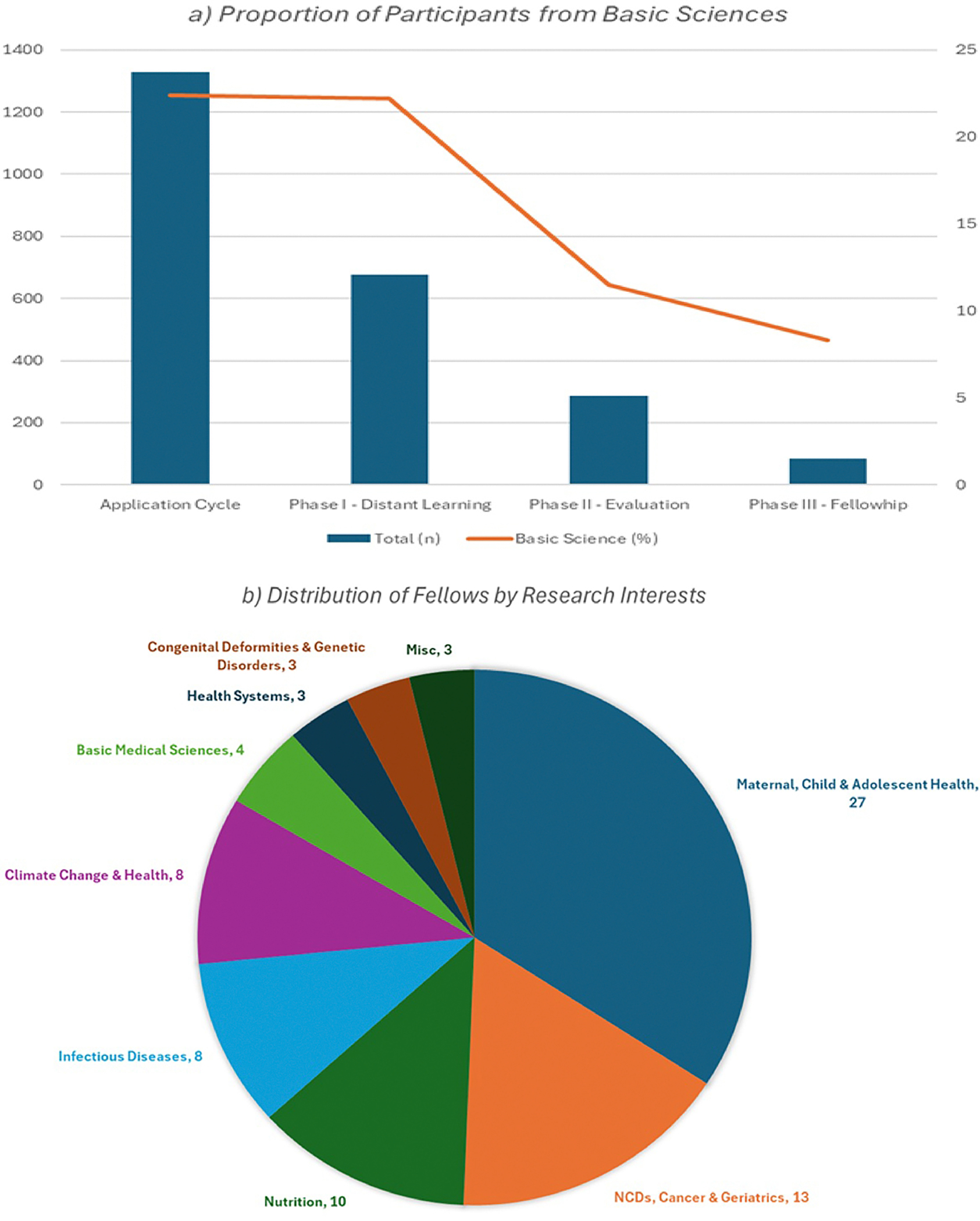
Proportion of participants from basic sciences & the distribution by research interests. (a) Proportion of Participants from Basic Sciences, (b) Distribution of Fellows by Research Interests

The SWIS Program actively invites female scientists from diverse academic backgrounds. Of the total applications received for the program (1329), 22.4% (297) had a basic science degree; Biological Sciences, Mathematics, Chemistry, and Physics [9]. Of these applicants, 677 were enrolled and in Phase I and 150 (22.2%) had a basic science background with minor variation across the first four cohorts:21.2% (28/132), 22.1% (41/186), 24.4% (44/180) and 20.7% (37/179), respectively. Nearly 60% of the total participants dropped out from Phase 1, highlighting the challenge of completing the self-paced coursework along with the existing work-life imbalance and this drop-out rate was higher for participants with a basic science degree being 78% (Figure 1a). Out of the total 84 participants selected for a fellowship as Phase III, only 7 (8%) had a basic science degree (Figure 1b).

The research interests of the fellows vary widely, encompassing diverse fields such as maternal and child health (MCH), nutrition, climate change, infectious diseases and cancer. This broad range reflects the multidisciplinary nature of the program, allowing participants to contribute to critical advances in their respective fields while benefiting from mentorship and capacity-building opportunities. Figure 1b represents the proportion of fellows according to their area of research interests.

Among the 84 Phase III fellows, 13 (15.5%) are conducting exclusively laboratory-based projects. All these projects focus on novel biomarker discovery for various health-related conditions. These are detailed in Table 1..

## Challenges and Lessons Learnt

The SWIS program addresses crucial research and academic gaps while offering an invaluable opportunity for female scientists from LMICs. It acts as a crucial steppingstone by strengthening the skills and expertise of early-to-mid-career female scientists and creating opportunities to advance into leadership roles and attain academic success. Witnessing the emergence of brilliant and innovative ideas from our cohort of fellows is incredibly inspiring, potentially leading to groundbreaking research and evidence generation for the global south. The concept of creating a platform for such exceptional individuals is remarkable. It enhances the participants’ capabilities, fosters a community of practice, and establishes a global network, facilitating collaboration on projects and the exchange of knowledge and ideas. The program builds upon the experience of established female researchers through a steering committee which was formed to identify challenges faced by women in research and guide the program structure.

Through the course of the program, we have encountered specific challenges in providing adequate support for the participants and fellows with basic science backgrounds. Initially, clinical courses were included in the core curriculum, which were challenging for those with a basic science background as the Phase I curriculum predominantly focused on epidemiology, biostatistics and research skills. To enhance inclusivity, the curriculum must be diversified to better accommodate female scientists from non-clinical disciplines.

The process of matching mentors to fellows that proposed laboratory-based projects in Phase III was challenging. Out of the 65 mentors matched across the four cohorts, nine have a basic science background. Similarly, finding appropriate peer-reviewers for the blind assessment of the fellows’ research proposals was a difficult task

Another major challenge has been navigating the complex administrative and regulatory processes often signifying the lack of transparent and well-defined institutional support mechanisms for research oversight and audit processes, which delayed the distribution of funds and project initiation. Some laboratory projects required costly equipment and materials, which were beyond the project funding limitations. Institutional investments in laboratory infrastructure and exploring collaborative funding mechanisms can help overcome these hurdles for which active engagement at the institutional level is essential.

## Way Forward

Patriarchal norms, inadequate maternity policies, and workplace biases exacerbate existing disparities [10]. Work-life balance remains a significant issue, often leading to non-tenure-track roles and slower career progression. [11]. Despite equal qualifications, men are more likely to be offered better opportunities and compensation [7]. Further, limited access to mentorship and professional networks restricts opportunities for collaboration and leadership roles [11]. Limited access to mentorship and professional networks further restricts women’s advancement and leadership opportunities, particularly in STEM fields [12], [13]. To address these inequities, the SWIS program operates within a broader institutional framework that confronts systemic barriers such as unequal grant access, gender stereotypes, and work-life imbalance.

SWIS is adapting to better support women in non-clinical fields by introducing virtual mentorship networks, discipline-specific online courses, and gender-sensitive application reviews. Comparative programs such as ARISE reinforce the value of mentorship, leadership development, and institutional accountability in advancing women’s careers in science [12]. As a way forward, the SWIS program plans to adopt frameworks like the Athena SWAN Charter[14], and UNESCO’s SAGA to support institutional reforms and reduce gender gaps in STEM [15].

To build sustainability, SWIS is fostering a collaborative network through peer learning, webinars, and annual conferences, aligned with SDG 17 (Partnerships for the Goals). Yet, further steps are required: curriculum restructuring, diversified funding through strategic partnerships, and collaboration with regional science bodies. Crucially, implementing monitoring and evaluation mechanisms will ensure data-driven refinement and long-term impact. By combining global frameworks with localized solutions, SWIS is emerging as a scalable, equity-driven model for strengthening women’s roles in science across LMICs.

## Figures and Tables

**Table 1:** Research projects focused on Diagnostics and Biomarkers

Area of interest	Research Title	Study Design
**Identifying Novel Bio-markers**		
Maternal & Reproductive Health	Metabolomic profile of human placentas from Small for Gestational Age (SGA), Large for Gestational Age (LGA) and normal pregnancies – An exploratory study.	Case-control
Maternal & Reproductive Health	The vaginal microbiome of women with preterm labor: A pilot study to establish the composition of vaginal microbes in preterm labor using DNA-based technology at Aga Khan Hospital.	Prospective Cohort
Maternal & Reproductive Health	Pregnancy maternal serum vitamin D levels & risk of pre-eclampsia: A case-control pilot study in Kenya.	Case-control
Maternal & Reproductive Health	A novel quick and cost-effective bedside bio-digital tool for the detection of non-functional Oxytocin to prevent postpartum hemorrhage (PPH): using Artificial Intelligence (AI) and embedded systems.	Human-Centered Design Science Research Methodology
Newborn & Child Health	Use of cranial ultrasonography to improve early diagnosis, management, and outcomes of meningitis in neonates with suspected sepsis in a setting with limited access to lumbar puncture at the University Teaching Hospital, Zambia.	Prospective Cohort
Climate Change & Respiratory Health	Investigating air pollution induced genome – Wide DNA methylation changes and respiratory health effects: A study in Karachi, Pakistan.	Cross-sectional
Infectious Diseases	Comparative analysis of EBV genotypic diversity, placental gene expression and salivary shedding in malaria-infected versus uninfected pregnant women.	Cross-sectional
Non-communicable Diseases	Glycemic control in children and adolescents with Type-1 diabetes: a comparison of flash glucose monitoring (FGM) and self-monitoring of blood glucose (SMBG).	Quasi-experimental
Cancer	Can primary and continuous breast cancer cell cultures from a Kenyan population be used to predict possible anti-cancer drug candidates?	Experimental
Cancer	Impact of cancer related distress estimated by distress thermometer on cerebral glucose metabolism and impact on chemotherapy response in lymphoma patients.	Longitudinal
Genetic Disorders	Genetic profiling of autoimmune diseases (Rheumatic Arthritis, Systemic Lupus Erythematosus)	To be determined
Geriatrics	Genetic & epigenetic markers of Alzheimer's disease.	To be determined
**Health impacts using Novel Biomarkers**		
Newborn & Child Health	Serum lipid metabolome and hepcidin-25 levels in children (24 – 59 months) with and without iron deficiency anemia	Case-control
Nutrition	Beyond nutrition: Investigating aflatoxin M1 in maternal milk and child growth outcomes.	Longitudinal
Climate Change & Infectious Diseases	Impact of climate change on HIV viral load and drug resistance in Senegal, West Africa.	To be determined
